# Mitophagy-related molecular signatures in ulcerative colitis revealed by machine learning and molecular dynamics

**DOI:** 10.3389/fgene.2026.1760869

**Published:** 2026-02-02

**Authors:** Yanru Han, Weihua Ren, Sujuan Li, Zhenxia Zhao, Zhiqiang Lin, Fucheng Zhao

**Affiliations:** Department of Integrated Chinese and Western Medicine, The First Affiliated Hospital of Henan Medical University, Weihui, China

**Keywords:** drug prediction, machine learning, mitophagy, molecular dynamic simulation, ulcerative colitis

## Abstract

**Introduction:**

Ulcerative colitis (UC) is a lifelong, chronic inflammatory disorder, characterized by recurrent and diffuse inflammation of the rectal and colonic mucosa. Increasing evidence suggests that impaired mitophagy contributes to immune dysregulation and epithelial injury in UC. However, the mitophagy-related molecular landscape and its therapeutic potential remain largely unexplored.

**Methods:**

Mitophagy-related genes (MRGs) were intersected with differentially expressed genes to identify UC-associated MRGs. Functional enrichment, immune infiltration, and consensus clustering analyses were performed to characterize molecular subtypes. Three machine learning methods were employed to identify diagnostic models. Candidate therapeutic agents were identified by the CMap database.

**Results:**

A total of 35 UC-associated MRGs were identified, enriched in cell activation, fatty acid metabolism, and the PPAR signaling pathway, revealing strong immunometabolic coupling in UC. Consensus clustering stratified UC patients into two subtypes: a metabolism-dominant subtype (C1) and an inflammation-activated subtype (C2). Three hub genes—CD55, CPT1A, and SLC16A1—were screened and validated as robust diagnostic markers. Drug prediction and molecular docking revealed strong binding between galunisertib and CD55, which was further validated by molecular dynamics simulations. *In vitro*, galunisertib significantly suppressed inflammatory cytokine release in LPS-induced UC cell models.

**Discussion:**

This study delineated the mitophagy-related molecular signatures of UC and identified CD55, CPT1A, and SLC16A1 as key biomarkers linking mitochondrial dysfunction, metabolic reprogramming, and immune activation. Furthermore, galunisertib was proposed as a potential therapeutic agent, providing a theoretical basis for UC therapy.

## Introduction

Ulcerative colitis (UC) is a lifelong, chronic inflammatory illness and a primary subtype of inflammatory bowel disease (Le Berre et al., 2023). Over the past few decades, the incidence of UC has increased markedly in developing countries, posing a substantial burden on patients’ quality of life. However, despite considerable advances in understanding UC pathophysiology, the exact etiology remains elusive (Ho and Theiss, 2022; de Sou et al., 2016). Therefore, many patients fail to achieve durable remission with existing therapies, underscoring the substantial heterogeneity of UC and the urgent need to find novel diagnostic and therapeutic biomarkers.

For the specific elimination of superfluous or malfunctioning mitochondria, mitophagy is an essential type of autophagy. There is growing evidence that autophagy uses the removal of malfunctioning mitochondria as a potent strategy to control the immune system (Xu et al., 2020). In addition to directly controlling mitochondrial antigen presentation, the process of mitophagy may limit the release of inflammatory cytokines. Emerging evidence highlights a close link between mitophagy and the pathogenesis of UC. Impaired mitophagy has been observed in UC patients and experimental models, leading to mitochondrial dysfunction, accumulation of reactive oxygen species (ROS), and excessive activation of inflammatory pathways such as the NLRP3 inflammasome (Lassen et al., 2014; Guo et al., 2014). Genetic variants associated with autophagy-related genes, including ATG16L1, IRGM, LRRK2, and PARK7, have been implicated in defective mitophagy, suggesting that disruption of mitochondrial quality control may contribute to intestinal inflammation and epithelial injury in UC (Kabi et al., 2012; Liu et al., 2013; Zhang et al., 2017). Moreover, pharmacological enhancement of mitophagy, such as through andrographolide, has been shown to attenuate colitis and colitis-associated carcinogenesis by suppressing inflammasome activation (Guo et al., 2014). These findings collectively indicate that mitophagy acts as a protective mechanism in UC by regulating mitochondrial homeostasis, limiting pro-inflammatory cytokine, and maintaining intestinal epithelial cell integrity. Understanding the molecular basis of mitophagy dysregulation in UC may therefore provide fresh perspectives on the etiology of the illness and point to possible targets for treatment.

To explore the hub mitophagy-related genes (MRGs) and potential mechanisms in UC, we employed an integrative bioinformatics strategy in this study. A UC diagnostic model was established using a comprehensive multi-algorithm machine learning framework. Single-cell analysis, immune correlation analysis, and functional enrichment analysis were conducted to investigate the biological characteristics and immunometabolic functions of the model genes.

## Methods

### Data processing and analysis

UC datasets were downloaded in the GEO, including GSE75214 (Vancamelbeke et al., 2017), GSE87466 (Li et al., 2018), GSE38713 (Planell et al., 2013), GSE48958 (Van der Goten et al., 2014). GSE87466 and GSE75214 were merged to constitute the training cohort. Batch effects within this merged training cohort were corrected using the “sva” package prior to feature selection. GSE38713 and GSE48958 were reserved as two completely independent external validation cohorts; they were not involved in the merging or batch correction process of the training set to ensure unbiased performance evaluation. Information on the clinical characteristics of the datasets is provided in Supplementary Table S1. 1715 MRGs (relevance score >1) were identified for subsequent analysis from the GeneCards (Hao et al., 2025).

### Functional enrichment analysis

The differentially expressed genes (DEGs) in UC were found by the “limma” program, which adjusted the P-value to less than 0.05 and met the requirements of |log2 FC| > 1. The PPI network was constructed by the STRING and GeneMANIA (Szklarczyk et al., 2023; Franz et al., 2018). The functional enrichment was conducted by “org.Hs.eg.db” package.

### Choosing feature genes and building diagnostic models

To build a molecular model for UC, three machine learning algorithms were conducted. These candidate genes were subjected to LASSO regression analysis using the “glmnet” software with 10-fold cross-validation to determine the ideal λ value (Wong et al., 2023; Li et al., 2025). The “e1071”and “caret” packages were used to conduct SVM-RFE analysis with a radial-basis-function kernel and 10-fold cross-validation (Shi et al., 2023). The “randomForest” program conducted the RF analysis (ntree = 500), and the variable importance ranking was based on MeanDecreaseGini (Xiao et al., 2024). Venn diagrams were used to identify genes common to all three models.

### Establishment of the UC cell model

An *in vitro* UC model was established by stimulating Caco-2 cells with LPS (1 μg/mL) for 24 h (Wang et al., 2025). To evaluate the therapeutic effect of LY2157299 (Beyotime, Shanghai, China) on UC, the UC cell model was treated with 10 μM LY2157299 (Zhu et al., 2024). IL-1β and IL-6 were measured to assess both the establishment of cell model and the therapeutic efficacy of LY2157299. The expression of model genes in the UC model was defined by qPCR. The primers are as follows: CD55, (forward) CCC​TCA​AAC​AGC​CTT​ATA​TCA​CTC and (reverse) AAT​ATG​CCA​CCT​GGT​ACA​TCA​ATC; CPT1A, (forward) GTA​TCT​ACA​GTC​GGT​GAG​GC and (reverse) GGA​TAT​ACA​GCA​GAT​CCA​TG; SLC16A1, (forward) GTG​GCT​TGA​TTG​CAG​CTT​C and (reverse) TGG​TCG​CCT​CTT​GTA​GAA​ATA​C; IL-1β, (forward) CTC​TCT​CCT​TTC​AGG​GCC​AA and (reverse) GAG​AGG​CCT​GGC​TCA​ACA​AA; IL-6, (forward) GTAGCCGCCCCACACAGA and (reverse) CAT​GTC​TCC​TTT​CTC​AGG​GCT​G. The comparison between the two groups was conducted using t-test, and *P* < 0.05 was statistically significant.

### GSVA and immune infiltration analysis

To explore the functional relevance of hub genes in UC, GSVA was applied across all UC samples (Hanzelmann et al., 2013). Immune cell infiltration was evaluated by the CIBERSORT method, quantifying the relative abundance of 22 immune cell subtypes per sample (Kim et al., 2021).

### Single-cell expression patterns and transcriptional regulatory analysis

Upstream transcription factors (TFs) regulating the hub genes were predicted by the TFTF platform (Wang, 2024), integrating three tools: GTRD (Kolmykov et al., 2021), ChIP_Atlas (Zou et al., 2024), and KnockTF (Feng et al., 2024). Single-cell expression patterns of model genes in normal colonic tissues were analyzed by the HPA database (Zhong et al., 2023).

### Computational drug prediction and protein-ligand interaction analysis

The CMap database (https://clue.io/) was used to screen candidate small molecules in order to find possible therapeutic drugs for UC (Zhou et al., 2023). The 3D structure of the CD55 protein was retrieved from the RCSB Protein Data Bank (PDB ID: 1h03). The structure of the candidate drug was retrieved from the PubChem database. Docking simulations were conducted by the CB-Dock2 (Liu et al., 2022). Subsequently, the MD simulation was performed using GROMACS (Li et al., 2024). The protein topology was generated using the CHARMM36 force field. The system was solvated in a cubic box using the TIP3P water model and neutralized with appropriate counter-ions. The equilibration process involved two steps: a 100 ps NVT ensemble to stabilize the temperature at 300 K (V-rescale thermostat) and a 100 ps NPT ensemble to maintain the pressure at 1 bar (Parrinello-Rahman barostat). Finally, a 100 ns production run was conducted.

## Results

### Molecular and immunological characteristics of UC patients

**FIGURE 1:**
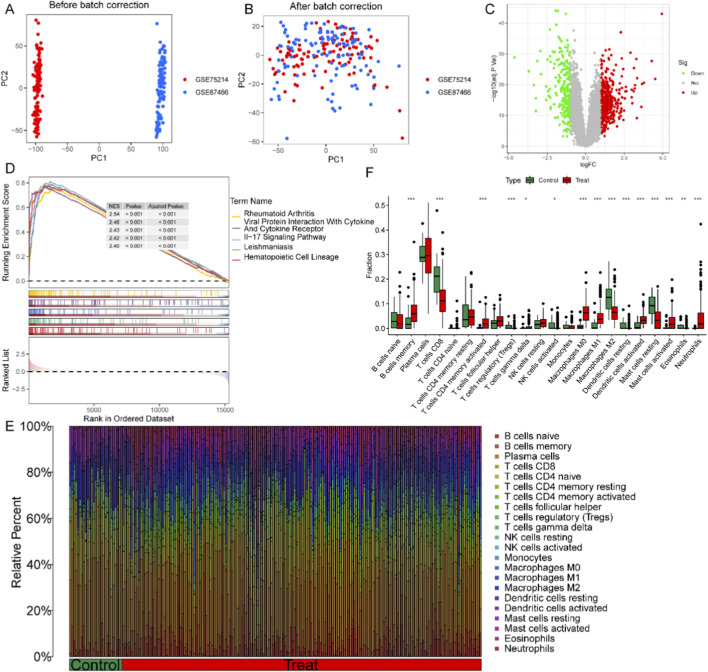
Molecular and immunological characteristics of UC patients. **(A,B)** PCA before and after batch correction. **(C)** Volcano plot of DEGs in UC. **(D)** Pathways upregulated in UC samples. **(E,F)** Comparison of immune cell infiltration between UC and normal groups. (*p < 0.05, **p < 0.01, and ***p < 0.001).

First, we merged the GSE87466 and GSE75214 datasets and performed batch effect correction. (Figures 1A,B; Supplementary Figure S1A,B). Ultimately, 695 DEGs were found and visualized (Figure 1C). To explore molecular differences between UC and normal samples, GSEA on the transcriptome profiles of the two groups was performed. UC samples were significantly enriched in viral protein interaction with cytokine and cytokine receptor, Il-17 signaling pathway, hematopoietic cell lineage (Figure 1D), suggesting the central role of aberrant immune activation in UC pathogenesis. The results of the CIBERSORT show that UC patients’ B cells memory, T cells CD4 memory activated, macrophages M0 and M1, dendritic cells activated, neutrophils were significantly upregulated, while T cells CD8, Tregs, and macrophages M2 were the opposite (Figures 1E,F).

### Differential expression analysis of MRGs

**FIGURE 2:**
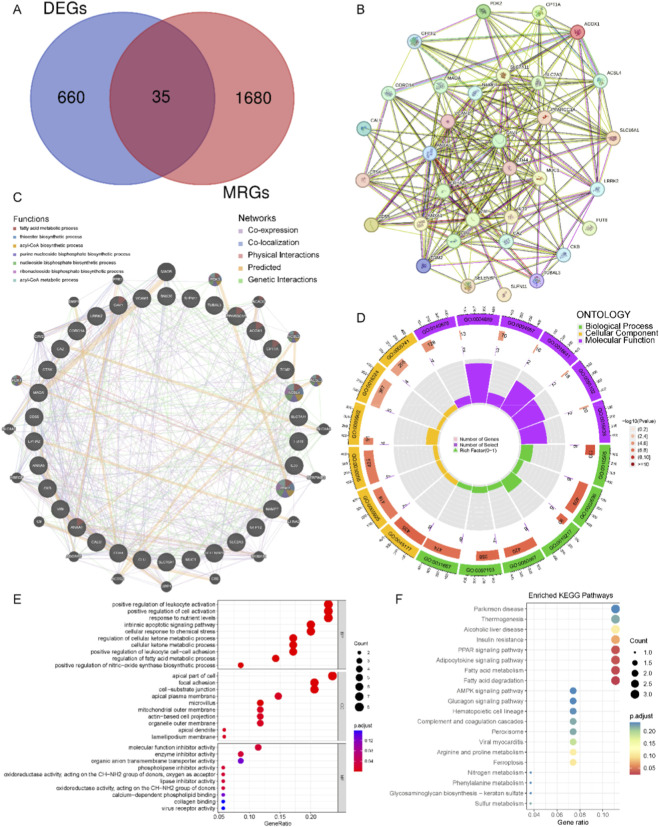
Identification of the MRGs in UC. **(A)** Venn diagram of intersected DEGs and MRGs. **(B)** PPI network of 35 common genes. **(C)** GeneMANIA analysis of 35 common genes. **(D–F)** Functional enrichment of 35 common genes.

The Venn diagram shows that a total of 35 MRGs were differentially expressed in UC (Figure 2A). Analysis based on the STRING and GeneMANIA databases revealed extensive interactions, co-expression, and colocalization among these genes, with significant enrichment in pathways related to fatty acid metabolism, acyl-CoA metabolism, and nucleotide metabolism (Figures 2B,C). Functional enrichment revealed that they were enriched in the positive regulation of cell activation, fatty acid metabolism, adipocytokine and PPAR signaling pathway (Figures 2D–F).

### MRG-related clusters in UC

**FIGURE 3:**
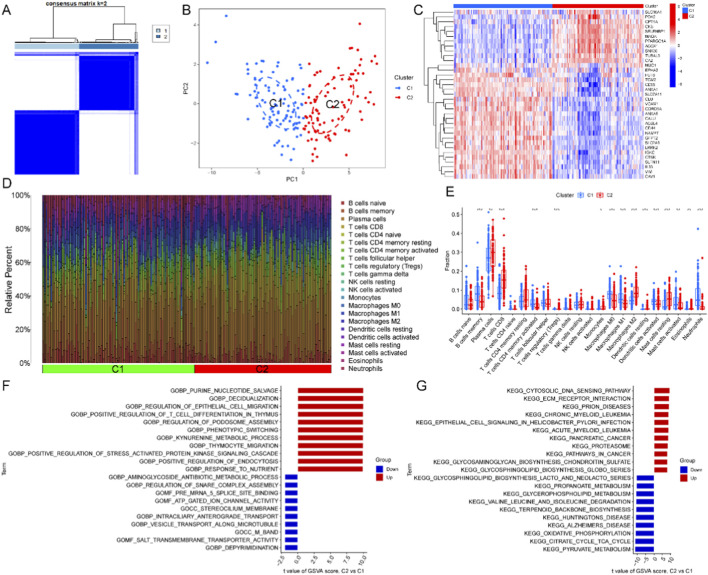
Two MRG-related molecular subtypes of UC. **(A)** Consensus clustering matrix with k = 2. **(B)** PCA showing the subtype distribution. **(C)** Expression profiles of UC-MRGs across the two identified clusters. **(D,E)** Immune characteristics of different molecular subtypes. **(F,G)** Differences in functional enrichment between two subtypes. (*p < 0.05, **p < 0.01, and ***p < 0.001).

A consensus clustering method was used to categorize UC samples into two clusters according to 35 UC-MRGs (Figures 3A–C). And C1 had higher concentrations of B cells, T cells with CD4 memory activated, macrophages (M0 and M1), activated dendritic cells, activated mast cells, and neutrophils. The plasma cells, T cells CD8, M2 macrophages, and mast cells resting were more enriched in C2 (Figures 3D,E). GSVA revealed that C2 (inflammation-activated subtype) was mainly enriched in immune and inflammatory pathways, such as the Proteasome, ECM-receptor interaction, and Cytosolic DNA sensing pathway, indicating enhanced immune activation and signaling activity (Figures 3F,G). In contrast, C1 (metabolism-dominant subtype) was primarily enriched in energy metabolism pathways, including the citrate cycle, oxidative phosphorylation, and pyruvate metabolism, vesicle transport along microtubule, and ATP-gated ion channel activity, suggesting active mitochondrial metabolism and enhanced cellular transport (Figures 3F,G).

### Using three machine learning algorithms to select biomarkers for UC

**FIGURE 4:**
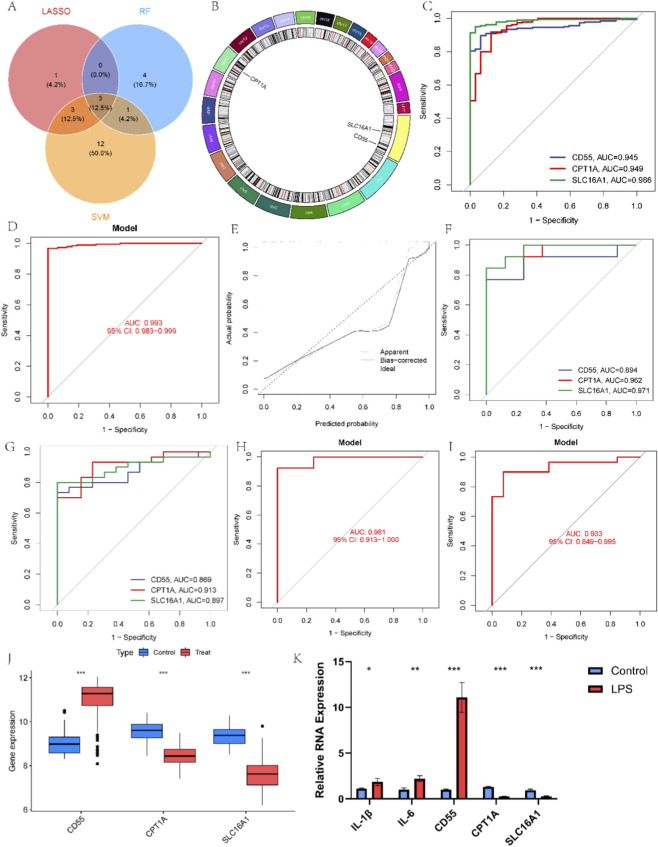
Diagnostic value of the model for UC. **(A)** Venn diagram of intersected genes. **(B)** Chromosomal localization of three model genes. **(C,D)** ROC for the individual genes and model in the training set. **(E)** Calibration curve for the model in the training set. **(F–I)** ROC for the individual genes and model in two individual test sets. **(J)** The level of the model genes in UC and normal samples. **(K)** The level of IL-1β, IL-6, and model genes in cell model.

To identify characteristic genes of UC and eliminate non-essential variables, LASSO, SVM-RFE, and RF were applied. LASSO regression initially selected seven candidate genes (Supplementary Figures S2A,B). SVM-RFE identified a set of 19 candidate genes based on recursive feature elimination (Supplementary Figures S2C,D). RF analysis quantified gene importance, selecting 8 genes with importance scores >2 as key features (Supplementary Figures S2E,F). Finally, we identified three core model genes—CD55, CPT1A, and SLC16A1—for further analysis (Figure 4A), while chromosomal mapping shows their genomic locations (Figure 4B). These three genes demonstrated excellent predictive performance (Figure 4C), while the three-gene model exhibited superior performance (AUC = 0.993, 95%CI: 0.983–0.999, precision = 1, recall = 0.967) (Figure 4D). Furthermore, the calibration curve demonstrated a high degree of consistency between the predicted probabilities and actual observations, indicating the robustness of the model (Figure 4E). To ensure the model’s reliability and rule out overfitting, we extensively evaluated its performance on two independent external validation sets. CD55, CPT1A, and SLC16A1 individually exhibited significant predictive power in both GSE48958 (Figure 4F) and GSE38713 (Figure 4G), confirming that the model captures genuine biological signals. More importantly, this model also performed exceptionally well on two independent external validation sets (GSE48958: AUC = 0.981, 95%CI: 0.913–1.000, precision = 1, recall = 0.923; GSE38713: AUC = 0.933, 95%CI: 0.851–0.992, precision = 0.964, recall = 0.9) (Figures 4H,I). The box plot displays the expression levels of the model genes, revealing that CD55 is significantly upregulated in UC, whereas CPT1A and SLC16A1 show the opposite trend (Figure 4J). Similarly, this result was validated on two external validation sets (Supplementary Figures S2G,H). Subsequent research further confirms that the expression of model genes in UC cell model is consistent with bioinformatics predictions (Figure 4K).

Furthermore, we evaluated the expression of three MRGs in colorectal cancer (CRC). We analyzed transcriptomic data from the TCGA and immunohistochemical results from the HPA database. The results showed that CD55 was upregulated in CRC, whereas CPT1A and SLC16A1 exhibited the opposite pattern (Supplementary Figure S3). These findings suggest that model genes may play a potential role in the carcinogenic transformation of UC.

### Functional enrichment analysis of CD55, CPT1A, and SLC16A1

To explore the functional mechanisms of three model genes in UC, GSVA was performed across all UC samples. In UC patients, the CD55 high-expression group showed enrichment in multiple metabolic and mitochondrial pathways, including pyruvate, butanoate, valine, tyrosine metabolism, and negative regulation of autophagy of mitochondrion (Supplementary Figures S4A,B). In UC patients, both CPT1A and SLC16A1 were significantly downregulated and negatively correlated with multiple energy metabolism-related pathways, including fatty acid metabolism, pyruvate metabolism, butanoate metabolism, peroxisome, and the PPAR signaling pathway (Supplementary Figures S4C–F). Specifically, CPT1A downregulation indicates suppressed fatty acid β-oxidation and mitochondrial energy generation, whereas reduced SLC16A1 expression suggests impaired lactate/pyruvate transport and disrupted metabolite exchange. Both were also negatively correlated with pathways involved in lipid and xenobiotic metabolism.

**FIGURE 5:**
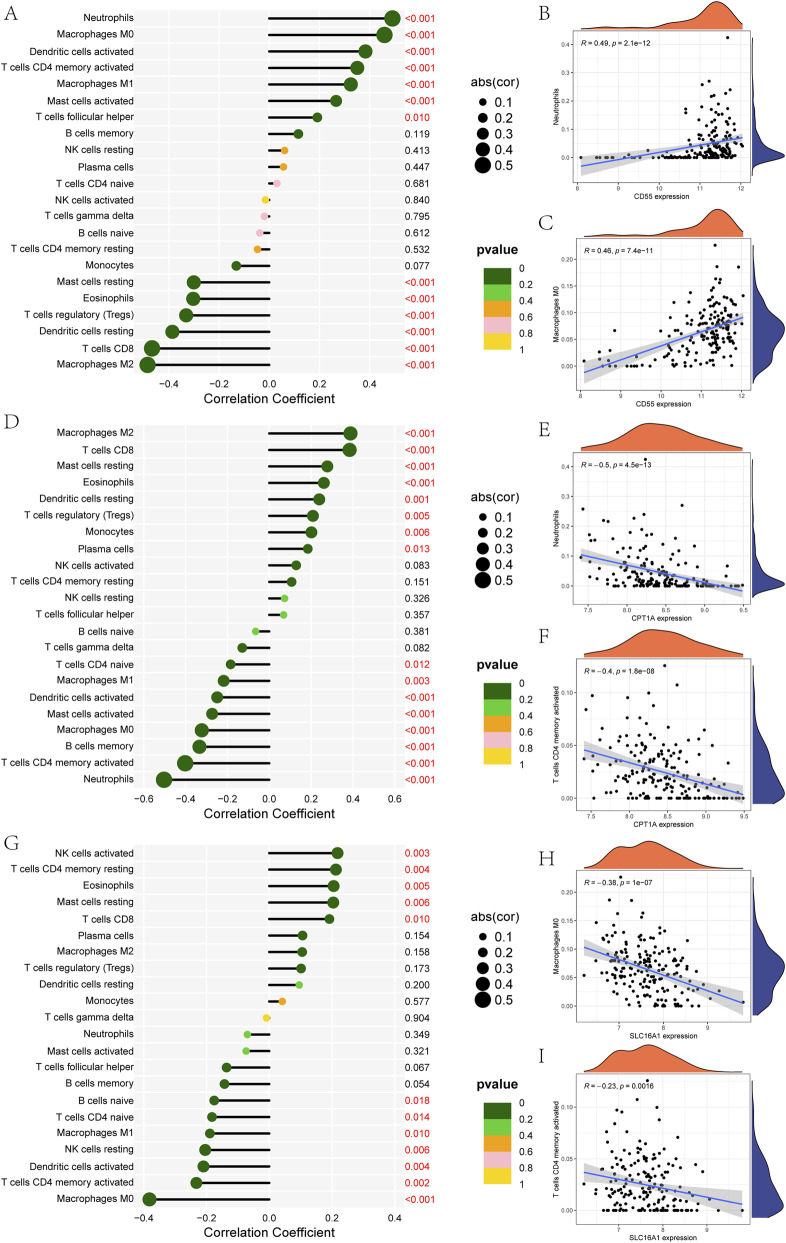
Immune features of CD55, CPT1A, and SLC16A1. **(A)** Correlations between CD55 and 22 types of immune cells. **(B)** Correlations between CD55 and neutrophils. **(C)** Correlations between CD55 and macrophages M0. **(D)** Correlations between CPT1A and immune cells. **(E)** Correlations between CPT1A and neutrophils. **(F)** Correlations between CPT1A and T cells CD4 memory activated. **(G)** Correlations between SLC16A1 and 22 types of immune cells. **(H)** Correlations between SLC16A1 and macrophages M0. **(I)** Correlations between SLC16A1 and T cells CD4 memory activated.

Immune infiltration analysis via CIBERSORT revealed distinct associations between CD55, CPT1A, and SLC16A1 and immune cell subtypes. CD55 was positively correlated with neutrophils and macrophages M0 (Figures 5A–C). CPT1A was negatively correlated with neutrophils and T cells CD4 memory activated (Figures 5D–F). SLC16A1 was negatively correlated with macrophages M0 and T cells CD4 memory activated (Figures 5G–I).

### TFs prediction and single-cell localization of hub genes

**FIGURE 6:**
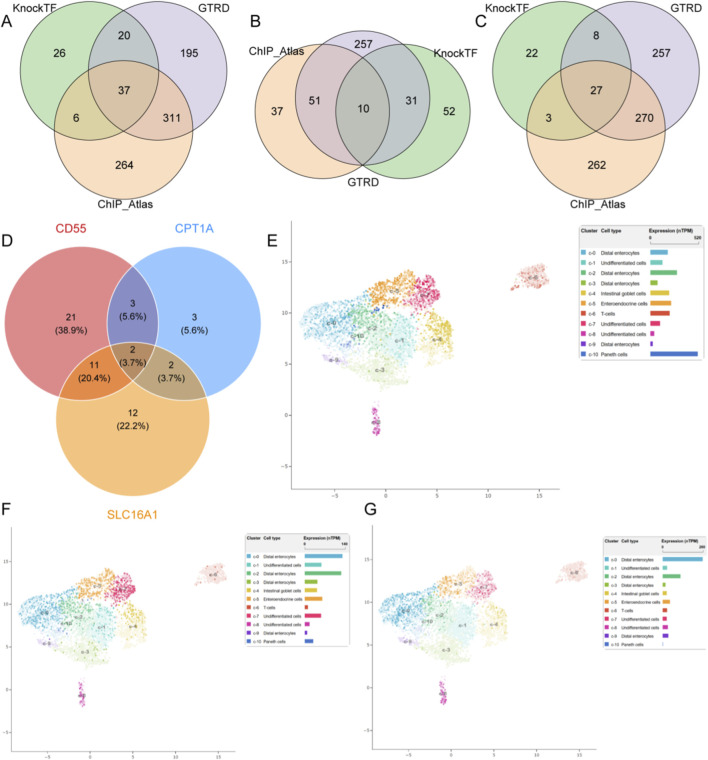
TF prediction and single-cell localization of the hub genes. TF prediction for **(A)** CD55, **(B)** CPT1A, and **(C)** SLC16A1 based on three databases. **(D)** The intersection of TFs for three genes. Single-cell expression patterns of **(E)** CD55, **(F)** CPT1A, and **(G)** SLC16A1.

Then, potential TFs regulating model genes were further identified based on multiple databases (Figures 6A–C). Finally, the intersection of TFs predicted by the three model genes revealed that SNAI2 and ESR1 may be their common upstream TFs (Figure 6D). To elucidate the roles of the three genes, we analyzed single-cell transcriptomic data of the colon, examining their expression patterns across different cell types. CD55 was more enriched in paneth cells (Figure 6E). CPT1A and SLC16A1 expression was more enriched in distal enterocytes (Figures 6F,G).

### Computational drug prediction and protein-ligand interaction analysis

**FIGURE 7:**
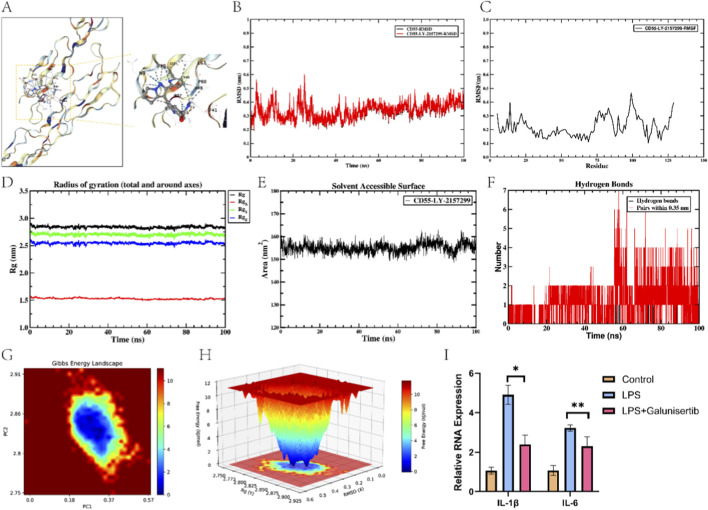
Computational study of CD55 binding with LY-2157299. **(A)** Docking structure of CD55-LY-2157299. **(B)** RMSD curves, **(C)** RMSF curves, **(D)** Rg curves, **(E)** SASA, **(F)** Hydrogen bond variations, **(G)** 2D FEL, **(H)** 3D FEL of the CD55-LY-2157299 complex. **(I)** Effects of LY-2157299 on IL-1β and IL-6 in an LPS-induced UC cell model.

In addition, we predicted the top five drugs for UC based on the CMap database. Given that CD55 was the only gene among the three model genes that was upregulated in UC, molecular docking was performed between the predicted drugs and CD55 using the CB-Dock2 platform. Among them (Supplementary Table S2), CD55 exhibited the best binding energy with galunisterib (LY-2157299), at −8.4 kcal/mol (Figure 7A). Supplementary Figure S4G showed the molecular docking results of galunisterib with the positive control TGF-β receptor I (−10.2 kcal/mol). Next, 100-ns MD simulations were conducted to assess the stabilization of the complex. The root mean square deviation (RMSD) profiles showed that the complex rapidly achieved equilibrium in the initial phase of the simulation and maintained overall stability thereafter (Figure 7B). Root mean square fluctuation (RMSF) suggested that, except for some loop regions, atomic fluctuations were generally mild (Figure 7C). Radius of gyration (Rg) curves and solvent-accessible surface area (SASA) further supported that the complex was compact and structurally stable overall (Figures 7D,E). Hydrogen bond analysis demonstrated that CD55-LY-2157299 maintained approximately 1-5 hydrogen bonds during the simulation (Figure 7F). The free energy landscape (FEL) analysis showed clear low-energy regions for the complex, suggesting high thermodynamic stability (Figures 7G,H). Finally, LY-2157299 was found to inhibit IL-1β and IL-6 in the LPS-induced UC model (Figure 7I).

## Discussion

UC is a chronic inflammatory disorder of the colorectum, in which dysregulated immune responses driven by disturbances in the gut microenvironment lead to recurrent tissue injury (Lu et al., 2026). Given the lack of reliable diagnostic biomarkers and effective therapeutic targets, the diagnosis and treatment of UC remain challenging. Mitophagy, a specialized form of autophagy, serves to identify, remove, and recycle damaged or depolarized mitochondria (Palikaras et al., 2018). It can also eliminate excess organelles, such as sperm mitochondria, after fertilization. To refill the mitochondrial pool with fresh, healthy organelles, mitophagy works in tandem with mitochondrial dynamics and biogenesis (Palikaras et al., 2015). Defective mitophagy results in the buildup of dysfunctional mitochondria, contributing to the development of various chronic diseases, including UC(4). Hence, a comprehensive understanding of the mechanisms regulating mitophagy in UC has significant clinical relevance.

The study first conducted a comprehensive analysis of the functional and immune characteristics of patients with UC. The results revealed enhanced cytokine signaling and excessive immune activation, indicating that the pathogenesis of UC involves not only localized intestinal inflammation but also systemic immune dysregulation and infection-like immune responses. Accumulating evidence suggests that the clearance of dysfunctional mitochondria through mitophagy is a crucial mechanism by which autophagy modulates immune homeostasis. Mitophagy may inhibit the release of inflammatory cytokines while also directly regulating mitochondrial antigen presentation and immune cell balance (Xu et al., 2020). To identify MRGs associated with UC progression, we identified 35 MRGs differentially expressed in UC. They were mainly involved in cell activation, fatty acid metabolism, adipocytokine signaling pathway, and PPAR signaling pathway. These results provide new insight into the mechanisms by which mitophagy influences intestinal inflammation and metabolic regulation. Furthermore, consensus clustering based on MRG expression stratified UC patients into different clusters: C1 (metabolism-dominant subtype) and C2 (inflammation-activated subtype). This molecular classification reveals the clinical heterogeneity of UC, wherein some exhibit pronounced immune activation and inflammatory infiltration, while others display metabolic dysregulation-dominant features.

Then we constructed a molecular diagnostic model for UC using multiple machine learning algorithms. Three model genes, CD55, CPT1A, and SLC16A1, were ultimately identified. Most notably, the model demonstrated strong diagnostic value across both the training set and two external independent test sets. In addition, we identified distinct expression patterns of CD55, CPT1A, and SLC16A1 in UC, revealing a potential link between immune activation and metabolic dysregulation. CD55 was significantly upregulated in UC tissues and positively correlated with neutrophil and M0 macrophage infiltration, suggesting that CD55 may contribute to innate immune activation and chronic inflammatory responses. The upregulation of CD55, a complement regulatory protein (Detsika et al., 2025), may reflect a compensatory immune activation under mitochondrial stress. Mitochondrial dysfunction is known to activate danger-associated molecular patterns and trigger complement and innate immune signaling (Supinski et al., 2020). Thus, elevated CD55 expression might represent a cellular attempt to counteract complement-mediated damage but may also contribute to persistent immune activation in the inflamed mucosa. In contrast, CPT1A and SLC16A1 were markedly downregulated in UC patients and negatively correlated with energy metabolism-related pathways, including Fatty acid metabolism, Pyruvate metabolism, Butanoate metabolism, Peroxisome, and PPAR signaling pathway. CPT1A is a pivotal enzyme controlling mitochondrial fatty acid β-oxidation and is essential for maintaining mitochondrial energy output and integrity (Schlaepfer and Joshi, 2020). Its downregulation in UC implies suppressed mitochondrial metabolism and impaired mitophagy, which may lead to the accumulation of dysfunctional mitochondria and excessive ROS, thereby fueling inflammatory responses. Similarly, the reduced expression of SLC16A1 may hinder lactate/pyruvate transport and limit the metabolic flexibility required for proper mitophagic flux and epithelial energy balance (Halestrap, 2013). Notably, both CPT1A and SLC16A1 expression were negatively correlated with activated CD4^+^ memory T cells, implying that metabolic suppression may accompany or drive sustained T-cell activation and inflammatory persistence. Together, these findings highlight a metabolic-immune axis in UC, where CD55-mediated immune activation coexists with CPT1A/SLC16A1-associated metabolic insufficiency. This reciprocal relationship between immune activation and metabolic exhaustion may underlie the chronic, relapsing nature of UC and suggests that modulating complement signaling or restoring mitochondrial and transport-mediated metabolism could represent promising therapeutic.

To investigate the potential upstream regulatory mechanisms of these model genes, TF analysis revealed SNAI2 and ESR1 as common candidate regulators of CD55, CPT1A, and SLC16A1. Both TFs have been reported to participate in epithelial homeostasis, inflammation, and metabolic reprogramming, suggesting a possible transcriptional link between immune activation and metabolic regulation in UC (Hevener et al., 2025; Zhang et al., 2025; Daniel et al., 2021; Li et al., 2014). Single-cell localization further supports cell-type-specific expression patterns: CD55 was mainly expressed in Paneth cells, suggesting a role in mucosal immune defense and complement regulation at the epithelial surface, whereas CPT1A and SLC16A1 were enriched in distal enterocytes, indicating their involvement in epithelial metabolic activity and nutrient processing. Together, these findings suggest that UC pathogenesis involves cell-type-specific transcriptional regulation linking immune activation, metabolic reprogramming, and epithelial barrier function, offering new insight into the coordinated control of immune-metabolic homeostasis in the intestinal mucosa.

Finally, we input the DEGs of UC into the CMap database for drug screening to find drugs that can effectively treat UC. Among the identified hub genes, CD55 was prioritized as the primary therapeutic target within a mitophagy-centered framework. First, mechanistically, CD55 acts as an “upstream gatekeeper” of mitochondrial homeostasis, regulating the initial complement-mediated injury that triggers mitophagy. Secondly, from a pharmacological perspective, unlike downregulated CPT1A and SLC16A1, CD55 is significantly upregulated in UC, providing a feasible surface target for inhibition. Drug prediction and molecular docking analyses showed that CD55 had high-affinity binding with galunisertib, findings further validated by MD simulations. Galunisertib (LY2157299) is a selective ATP-competitive inhibitor of transforming growth factor-β (TGF-β) receptor I and is currently the only known small-molecule inhibitor specifically targeting the TGF-β signaling pathway (Herbertz et al., 2015). The TGF-β pathway plays a dual role in maintaining intestinal homeostasis: under physiological conditions, it promotes mucosal repair and immune tolerance; however, under chronic inflammatory conditions, sustained activation of TGF-β signaling can induce fibrotic responses, epithelial-mesenchymal transition, and aberrant immune cell activation, thereby exacerbating tissue damage and chronic inflammation. Therefore, moderate inhibition of excessive TGF-β signaling may help alleviate intestinal inflammation and restore mucosal homeostasis. Consistent with our finding that galunisertib exhibits strong binding affinity with CD55, we propose a potential mechanistic convergence. Mechanistically, CD55 acts upstream of mitophagy by preventing complement-mediated mitochondrial injury (calcium overload and depolarization). Its upregulation in UC represents a targetable stress marker. We hypothesize that galunisertib may target this CD55-high microenvironment to modulate the immune-metabolic interface and facilitate the restoration of homeostatic mitophagy, rather than inhibiting CD55’s protective function. This hypothesis aligns with previous studies showing that galunisertib can attenuate inflammatory responses in diverse disease models (Liu et al., 2016; Wang et al., 2020). Our *in vitro* validation provided preliminary support for this potential: galunisertib treatment significantly suppressed the release of inflammatory cytokines in LPS-induced UC cell models. However, it must be explicitly stated that the interaction between CD55 and galunisertib identified in this study is currently based on computational prediction and molecular dynamics simulations. These mechanistic insights are hypothetical and exploratory in nature, serving as a theoretical basis that requires rigorous experimental validation in future *in vivo* and *in vitro* studies.

However, this study has some limitations. First, it was based on publicly available transcriptomic datasets, and sample size and population heterogeneity may affect the generalizability of the model. Second, due to the lack of detailed clinical metadata in the public datasets, we could not correlate the identified molecular subtypes with disease severity or therapeutic response. Future studies utilizing clinically annotated cohorts are required to validate whether these subtypes can effectively stratify patients for personalized therapy. In addition, although MD simulations have confirmed the structural stability of the CD55-galunisterib complex through RMSD and hydrogen bonding analysis, the precise thermodynamic affinity needs to be further quantitatively validated in future research. While we validated the expression of hub genes and the anti-inflammatory effect of galunisertib in an LPS-induced cell model, we did not directly assess mitophagic flux or mitochondrial membrane potential. Furthermore, no functional experiments targeting CD55 were performed to confirm its direct causal role in mitophagy regulation. Therefore, the link between CD55 inhibition by galunisertib and mitophagy modulation remains a bioinformatic prediction and a theoretical hypothesis. Our current experimental results support the drug’s anti-inflammatory potential, but the precise molecular mechanism requires rigorous verification in future studies.

## Data Availability

Publicly available datasets were analyzed in this study. This data can be found here: https://www.ncbi.nlm.nih.gov/gds (GSE75214, GSE87466, GSE38713, GSE48958).
