# Supplementary material for: Mitophagy-related molecular signatures in ulcerative colitis revealed by machine learning and molecular dynamics
Source: Front Genet. 2026 Feb 2;17:1760869. doi: 10.3389/fgene.2026.1760869 (PMC12906901; doi:10.3389/fgene.2026.1760869)
Supplement: Supplementary file 1 [file DataSheet1.pdf]

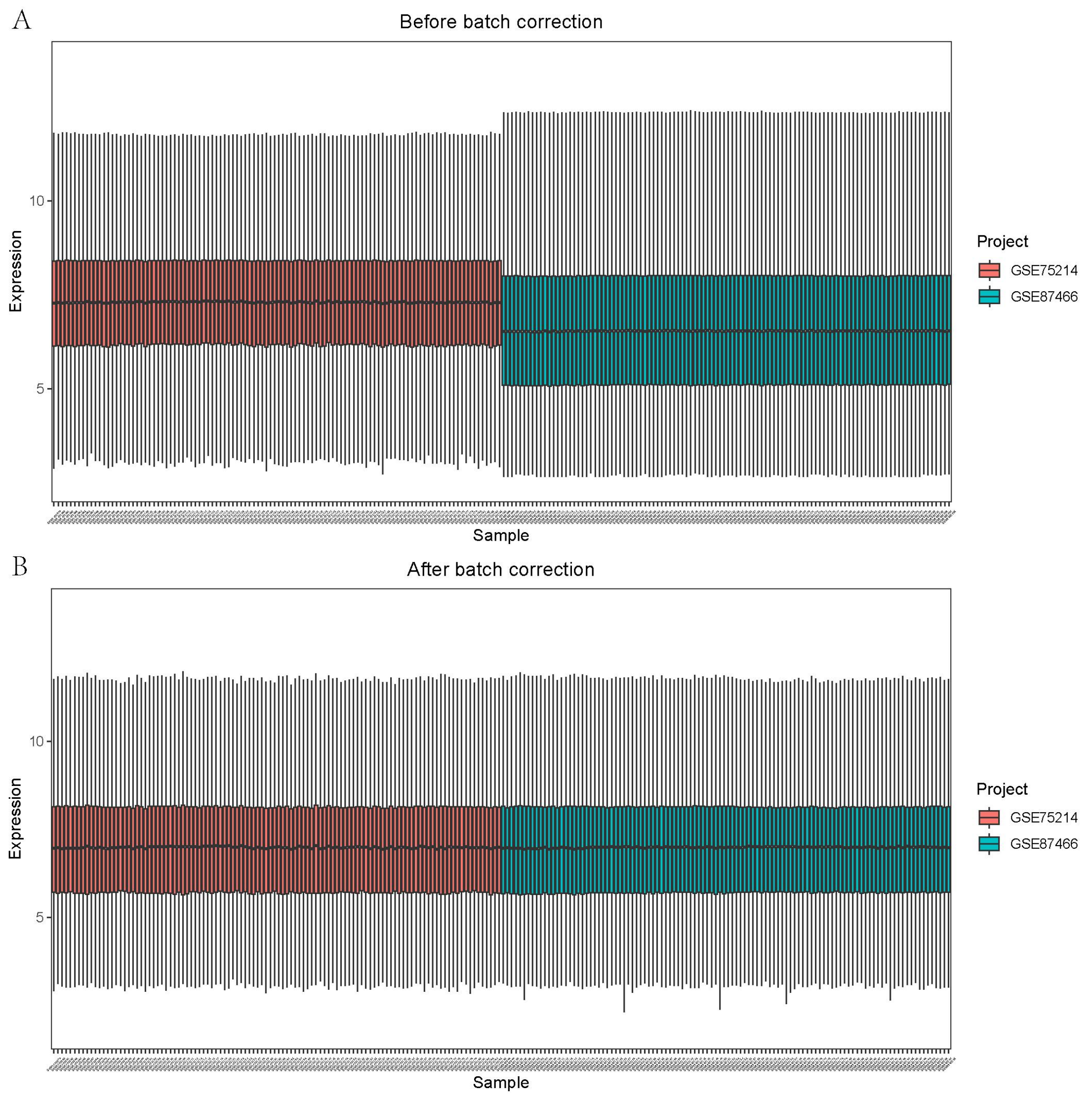

Fig. S1.Merging and calibration of datasets. (A-B) Boxplots prior to and following the UC dataset merger.

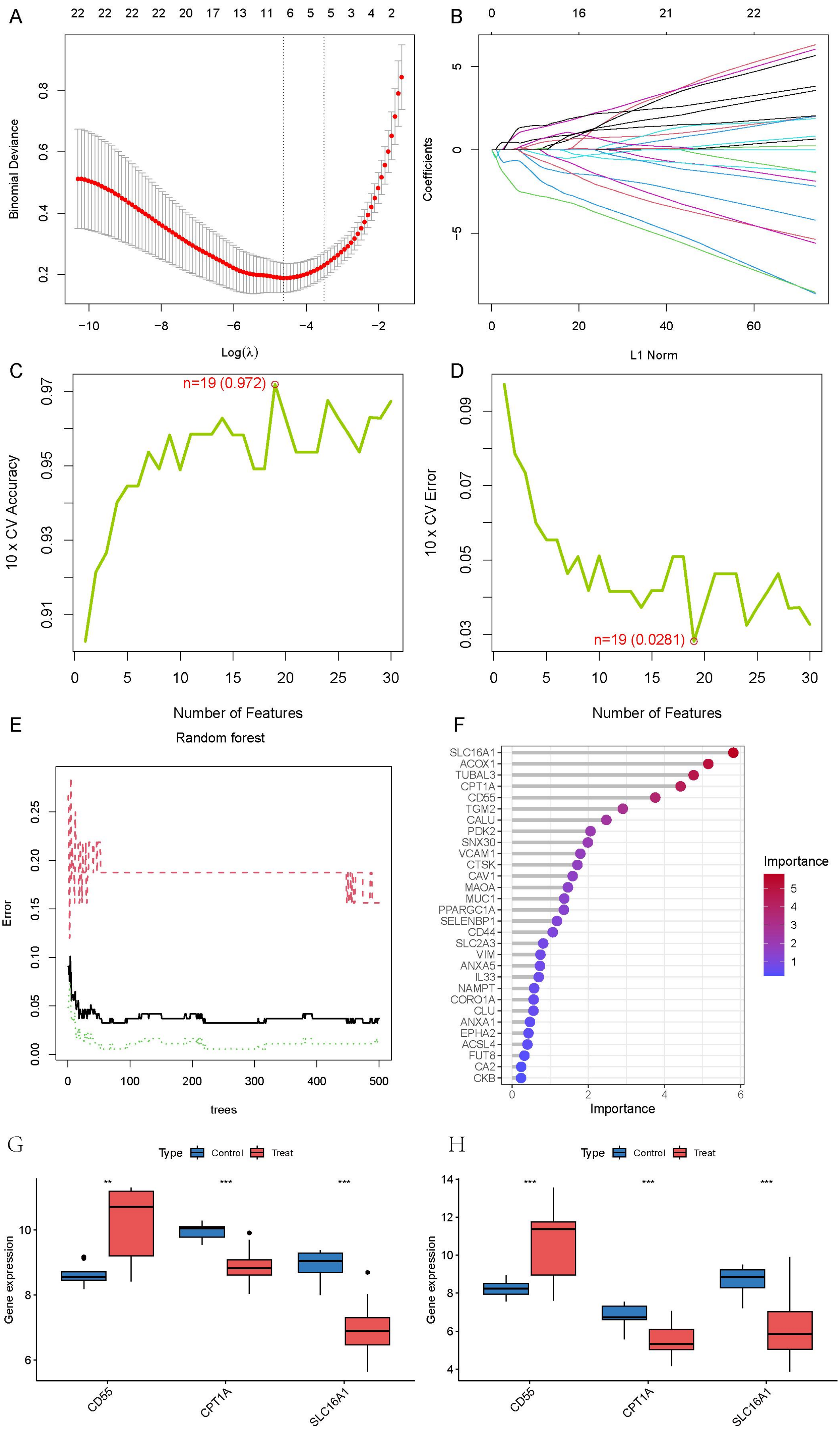
A
B
C
D
E
F
G
H
Binomial Deviance
Log(λ)
Coefficients
L1 Norm
n=19 (0.972)
10 x CV Accuracy
Number of Features
n=19 (0.0281)
10 x CV Error
Number of Features
Random forest
Error
trees
SLC16A1
ACOX1
TUBAL3
CPT1A
CD55
TGM2
CALU
PDK2
SNX30
VCAM1
CTSK
CAV1
MAOA
MUC1
PPARGC1A
SELENBP1
CD44
SLC2A3
VIM
ANXA5
IL33
NAMPT
CORO1A
CLU
ANXA1
EPHA2
ACSL4
FUT8
CA2
CKB
Importance
Type Control Treat
Gene expression
CD55
CPT1A
SLC16A1

Fig. S2. Construction of UC diagnostic models using three machine learning methods. (A-B) Seven genes identified by LASSO. (C-D) Nineteen genes selected by SVM-RFE. (E–F) Eight genes (importance score > 2) identified by RF analysis. (G-H) The expression of three model genes in two test sets.

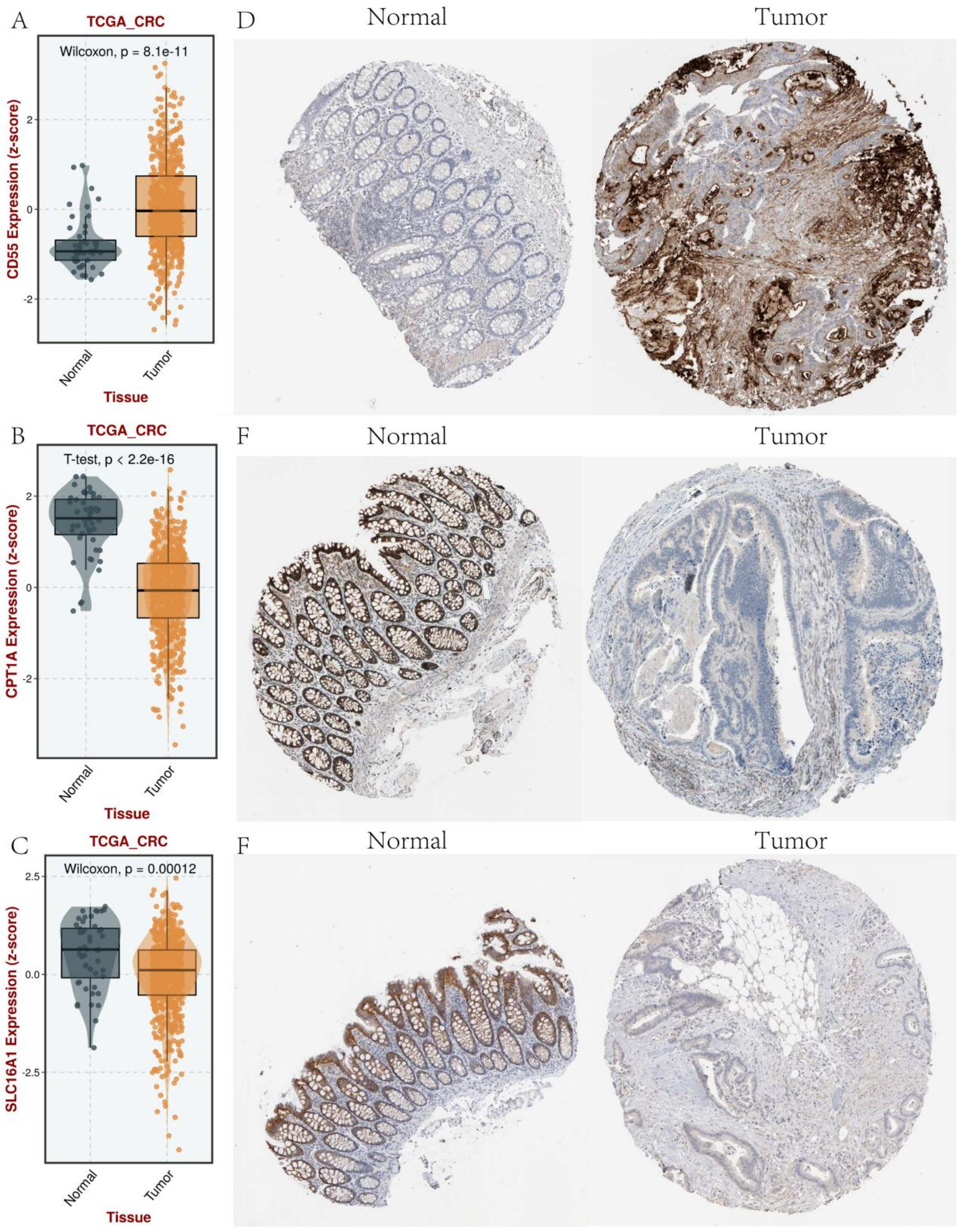

Fig. S3. Expression of three model genes in CRC. (A-C) Expression levels of three model genes in CRC tissues compared with adjacent normal tissues. (D-F) Representative immunohistochemistry staining of three model genes in paired para-carcinoma and CRC tissues.

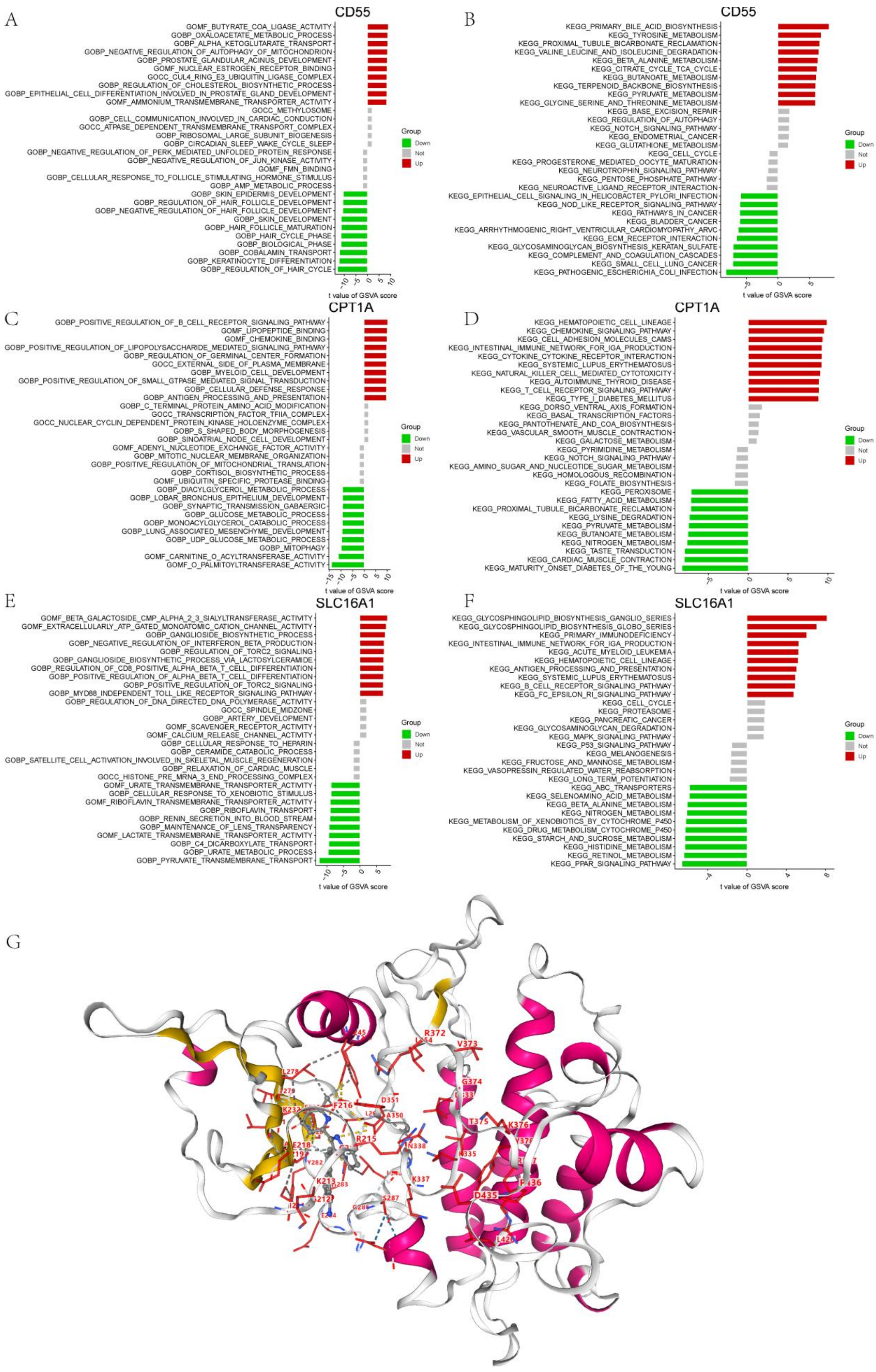

Fig. S4. GSVA of model genes. Comprehensive scoring of (A-B) CD55, (C-D) CPT1A and (E-F)

SLC16A1 using the GSVA to explore their potential molecular mechanisms in UC. (G) Docking structure of TGF - β receptor I ((PDB ID: 1py5)-LY-2157299

Supplementary Table 1. Information on the clinical characteristics of the samples used in the GEO dataset.

Dataset	UC samples	Normal samples
GSE87466	87 patients with UC	21 normal samples
GSE75214	97 patients with UC	11 normal samples
GSE38713	30 patients with UC	13 normal samples
GSE48958	13 patients with UC	8 normal samples

Supplementary Table 2. Molecular docking of CD55 and the top five drugs

Gene	Drug	free binding energy (kcal/mol)
CD55	ajmaline	-7.2
	flunisolide	-7.2
	levamisole	-5.6
	LY-2157299	-8.4
	testosterone	-7.1
